# Diversity patterns and drivers of soil microbial communities in urban and suburban park soils of Shanghai, China

**DOI:** 10.7717/peerj.11231

**Published:** 2021-04-15

**Authors:** Weiwei Zhang, Jigang Han, Haibing Wu, Qicheng Zhong, Wen Liu, Shanwen He, Lang Zhang

**Affiliations:** 1 Key Laboratory of National Forestry and Grassland Administration on Ecological Landscaping of Challenging Urban Sites, Shanghai Academy of Landscape Architecture Science and Planning, Shanghai, China; 2 Shanghai Engineering Research Center of Landscaping on Challenging Urban Sites, Shanghai, China

**Keywords:** Urban development, Microbial community composition, Molecular ecological network, Soil properties, Heavy metals

## Abstract

**Background:**

The rapid expansion of urbanization leads to significant losses of soil ecological functions. Microbes directly participate in key soil processes and play crucial roles in maintaining soil functions. However, we still have a limited understanding of underlying mechanisms shaping microbial communities and the interactions among microbial taxa in park soils.

**Methods:**

In this study, the community variations of bacteria and fungi in urban and suburban park soils were investigated in Shanghai, China. Real-time PCR and high-throughput Illumina sequencing were used to examine the microbial abundance and community composition, respectively.

**Results:**

The results showed that soil molecular biomass and fungal abundance in urban park soils were significantly higher than those in suburban park soils, while no significant difference was observed in the bacterial abundance between urban and suburban park soils. The alpha diversity of soil microbes in urban and suburban park soils was similar to each other, except for Chao1 index of fungal communities. The results of similarity analysis (ANOSIM) revealed remarkable differences in the composition of bacterial and fungal communities between urban and suburban park soils. Specifically, park soils in urban areas were enriched with the phyla Methylomirabilota and Verrucomicrobiota, while the relative abundance of Gemmatimonadota was higher in suburban park soils. Moreover, the fungal class Eurotiomycetes was also enriched in urban park soils. Compared with suburban park soils, nodes and average paths of the bacterial and fungal networks were higher in urban park soils, but the number of module hubs and connectors of the bacterial networks and negative interactions among bacterial taxa were lower. Compared with suburban park soils, Acidobacteriota bacterium and Mortierellomycota fungus played more important roles in the ecological networks of urban park soils. Soil available zinc (Zn), available nitrogen (N), pH, and total potassium (K) significantly affected fungal community composition in park soils in Shanghai. Soil available Zn was also the most important factor affecting the bacterial community composition in this study.

**Conclusion:**

There were significant differences in the soil molecular biomass, fungal abundance, and the community composition and co-occurrence relations of both soil bacterial and fungal communities between urban and suburban park soils. Soil available Zn played an important part in shaping the structures of both the bacterial and fungal communities in park soils in Shanghai.

## Introduction

Urbanized areas have been rapidly expanding for decades (Grimm et al., 2008). Urban park soil, which is neither sealed nor compacted, represents one of the most important components of urban ecosystems and provides essential ecosystem services, such as nutrient cycling and carbon sequestration (Edmondson et al., 2014; Setälä et al., 2016), pollutant degradation (Escobedo, Kroeger & Wagner, 2011), and storm water interception and purification (Taka et al., 2017; Valtanen, Sillanpää & Setälä, 2015). Previous studies have reported that urban development has significant effects on the physical and chemical properties of urban soils (Ghosh, Scharenbroch & Ow, 2016; Li et al., 2011; Park et al., 2010; Yan et al., 2018); however, microbes in urban soils, especially in urban park soils, have received relatively little attention (Bünemann et al., 2018; Li et al., 2018).

Microbes are directly linked to key soil processes and play crucial roles in maintaining ecosystem services (Barrios, 2007; Haritash & Kaushik, 2009; Ledin, 2000; Li et al., 2009). Moreover, microbial communities in park soils may also have implications in human health because soil microbes are an important source of airborne microbial diversity (Bertolini et al., 2013; Hanski et al., 2012; Li et al., 2018). A growing number of studies have investigated microbes in park soils (Francini et al., 2018; Hui et al., 2017; Ramirez et al., 2014; Wang et al., 2018; Wang, Wu & Kumari, 2018; Xu et al., 2014). Some studies have shown that soil bacterial diversity in highly urbanized areas was significantly lower than that in suburban areas (Xu et al., 2014; Yan et al., 2016). In contrast, Ramirez et al. (2014) found similar bacterial diversity between soil from Central Park in New York City and the global data set (including arctic, tropical and desert soils). Recently, a study conducted in Shanghai, China showed that the dominant bacterial phyla in urban park soils were Proteobacteria and Acidobacteriota, which was in agreement with studies on bacterial communities from agricultural or forest soils (Wang, Wu & Kumari, 2018). It should be noted that these studies mainly focused on soil bacteria. Studies that simultaneously account for both bacteria and fungi within urban ecosystems are still lacking, even though they are both important for plant growth and soil ecological function. Moreover, to our knowledge, there has been no research exploring the co-occurrence patterns and interactions among taxa within the microbial community in park soils.

Driving factors of soil microbes have received substantial interest in natural ecosystems, however, few studies have focused on how soil microbes are influenced by biotic and abiotic factors in park soils (Francini et al., 2018; Hu et al., 2018; Hui et al., 2017; Wang et al., 2018; Xu et al., 2014; Yan et al., 2016). Bacterial diversity and composition have been shown to be affected by soil pH, moisture, and the carbon:nitrogen (C:N) ratio in urban parks, which is similar to the effects of these variables in natural ecosystems (Wang et al., 2018; Xu et al., 2014; Yan et al., 2016). In addition, urban park soil is generally enriched with heavy metals because of anthropogenic activities, such as the combustion of fossil fuels (Setälä et al., 2017; Yesilonis, Pouyat & Neerchal, 2008). It has been reported that heavy metal concentration was the main factor influencing microbial biomass and microbial community functional diversity in urban areas (Zhao et al., 2013). However, there are still not enough studies to clarify how these abiotic variables shape soil bacterial and fungal communities in urban parks and their relative importance in determining microbial community assemblages. A better understanding of the distributional patterns and major drivers of both bacteria and fungi in urban park soils is therefore important for elucidating microbial processes and for maintaining urban ecosystem functions.

Our main aim was to investigate soil bacterial and fungal communities in park soils and identify the primary driving factors that structure soil microbial communities. For this purpose, we investigated the abundance and composition of the bacterial and fungal communities in 20 park soil samples in urban and suburban areas in Shanghai. We hypothesized that (1) the abundance and diversity of the microbial communities in urban park soils may be significantly lower than those in suburban park soils; (2) the composition and network architecture of the microbial communities in urban park soils were significantly different from those in suburban park soils; and (3) the major factors driving the bacterial and fungal communities would differ due to the differences in their metabolic processes.

## Materials and Methods

### Study area and sampling

The metropolitan city of Shanghai was selected as the study area (31.14°N, 121.29°E). Adjacent to the Pacific Ocean, this area has a subtropical monsoon climate. The mean annual temperature and precipitation are 15.8 °C and 1,122 mm, respectively (Shi et al., 2008). The development of ring roads is a feature of urban sprawl in Chinese cities (Yan et al., 2016). Currently, four ring roads have been developed in Shanghai, including the Inner Ring Highway, the Middle Ring Highway, the Outer Ring Highway, and the Suburb Ring Expressway. The area inside of the Inner Ring Highway was considered as the urban area. The area outside of the Suburb Ring Expressway was considered as the suburban area. According to the location relative to the city ring road, the park is classified as “urban” or “suburban”. Ten monospecific stands (20 × 20 m) of *Cinnamomum camphora* (L.) Presl, were randomly chosen in urban and suburban parks, respectively (Table 1). The *Cinnamomum camphora* monocultures were at the age of 20 ~ 25 years old. Plants in all the studied stands were similar. Besides *Cinnamomum camphora*, stands also contained a few *Ophiopogon japonicus* (Linn. f.) Ker-Gawl in the herbage layer. Soil samples were collected in November 2017. In each stand, nine random soil samples (0–20 cm) were collected using a soil corer (2.5 cm diameter) and then mixed thoroughly and pooled into one composite sample. Soil was sieved through a 2-mm mesh immediately after sampling. Sampling and sieving of soil samples were carried out within 20 min. Each soil sample was divided into two subsamples. One subsample was air-dried to determine the chemical properties of the soil, and the other subsample was stored at −80 °C for DNA extraction.

**Table 1:** General attributes of the stands in this study.

Sample ID	Name of Park	Latitude	Longitude	Mean tree height (m)	Diameter at breast height (cm)	Tree density (stem hm ^−2^)
Urban 1	Shiji Park	31°12′54.55443″	121°32′29.59623″	14.4	13.3	1,050
Urban 2	Guangchang Park	31°13′38.06663″	121°28′16.48950″	10.0	12.0	862
Urban 3	Guangchang Park	31°13′36.48693″	121°28′05.11618″	9.9	12.9	766
Urban 4	Renmin Park	31°14′03.22925″	121°28′01.35861″	11.0	13.4	833
Urban 5	Shiji Park	31°12′55.65345″	121°32′51.89794″	15.6	13.8	1,175
Urban 6	Shiji Park	31°13′11.82202″	121°33′18.67060″	14.9	12.2	925
Urban 7	Jingnan Park	31°14′14.08009″	121°32′54.90683″	9.9	11.7	1,090
Urban 8	Jingnan Park	31°14′15.59368″	121°32′55.81082″	9.5	11.8	964
Urban 9	Mengqing Park	31°15′09.26895″	121°26′11.83790″	10.1	13.5	1,025
Urban 10	Luxun Park	31°16′14.51271″	121°28′43.13616″	13.6	14.1	825
Suburban 1	Haiwan Forest Park	30°52′03.90882″	121°41′23.72492″	11.9	11.4	650
Suburban 2	Haiwan Forest Park	30°52′05.02758″	121°41′38.02877″	12.8	11.7	1,125
Suburban 3	Haiwan Forest Park	30°51′51.91215″	121°41′58.22355″	11.7	13.2	807
Suburban 4	Binhai Forest Park	30°58′20.40587″	121°54′23.35727″	10.0	11.5	825
Suburban 5	Binhai Forest Park	30°58′04.89937″	121°54′26.69332″	9.8	12.2	975
Suburban 6	Binhai Forest Park	30°57′42.28336″	121°54′33.31795″	11.9	12.2	1,050
Suburban 7	Xincheng Park	31°37′21.29734″	121°25′18.67193″	10.4	12.7	1,191
Suburban 8	Xincheng Park	31°37′18.08624″	121°25′13.42979″	9.5	12.6	795
Suburban 9	Dongping Forest Park	31°40′47.06888″	121°28′39.31260″	11.8	13.7	864
Suburban 10	Dongping Forest Park	31°41′33.20447″	121°28′23.03739″	11.5	11.3	1,120

### Soil property measurements

Soil pH was measured in a 1:2.5 soil:water (w/v) mixture using a pH meter. Soil organic carbon (SOC) was analyzed using the dichromate oxidation method (Cui et al., 2019). Total nitrogen (N) was measured by the Kjeldahl method (Bremner & Mulvaney, 1982). Total phosphorus (P) was determined following H_2_SO_4_–HClO_4_ digestion (Olsen & Sommers, 1982). Total potassium (K) was determined by flame photometry after digestion in nitric acid, perchloric acid and hydrofluoric acid. Soil available N was measured by the alkali hydrolysis and diffusion method (Cornfield, 1960). Available P, K, copper (Cu), lead (Pb), cadmium (Cd), chromium (Cr), zinc (Zn), nickel (Ni), and arsenic (As) were extracted by AB-DTPA (ammonium bicarbonate-diethylenetriamine pentaacetic acid) (Soltanpour & Schwab, 1977). The available concentrations of K, Cu, Pb, Cd, Cr, and Zn were measured using ICP-OES (7000DV; Optima, Brookfield, WI, USA), and the available concentrations of P, Ni, and As were measured using ICP-MS (NexION 300X; PerkinElmer, Inc., Waltham, MA, USA).

### DNA extraction

Total genomic DNA was extracted from 0.5 g soil material using a FastDNA® SPIN Kit for soil (MP Biomedicals, Santa Ana, CA, USA) according to the manufacturer’s instructions. DNA concentration was determined on a Nanodrop 2000 UV-Vis Spectrophotometer (Thermo Scientific, Waltham, MA, USA). DNA quality was checked by 1% agarose gel electrophoresis and stored at −80 °C until use.

### Real-time PCR assay

Quantification of the bacterial 16S rRNA and fungal 18S rRNA gene was performed on a Light Cycler® 96 System (Roche, Mannheim, Germany). The bacterial 16S rRNA gene was quantified using the primers 338F and 518R (Park & Crowley, 2006), and the fungal 18S rRNA gene was quantified using the primers SSU-0817F and SSU-1196R (Borneman & Hartin, 2000). Real-time PCR assays were carried out in a 20 μl reaction volume using SYBR® Premix Ex Taq™ II (Takara, Kusatsu, Japan). Each 20 μl amplification reaction system contained 10 μl of SYBR Green PCR Mix, 0.2 μM of forward and reverse primers, and approximately 8 ng extracted DNA. The cycling conditions were as follows: 95 °C for 5 min; 40 cycles of 95 °C for 10 s, 55 °C for 30 s, and 72 °C for 40 s. Product specificity was confirmed by melting curve analysis and gel-electrophoresis of the amplified fragments. For standard curve preparation, amplicons of each targeted gene were cloned into the pMD™18-T vector (Takara, Kusatsu, Japan). Plasmids containing the target genes were extracted and used as standards for real-time PCR. The sequences of target genes used to construct the standard curves of bacteria and fungi have high sequence similarity (100%) with the members of the order Acidobacteriales and genus *Neobulgaria premnophila*, respectively. Plasmid DNA concentration was determined on a Nanodrop 2000 UV-Vis Spectrophotometer (Thermo Scientific, Waltham, MA, USA). To generate standard curves, three independent experiments were carried out using ten-fold serial dilutions of linearized plasmids. Amplification efficiencies for the bacterial and fungal assays were 103% and 93%, respectively. Data are shown as rRNA gene copy numbers per gram of dried soil.

### Illumina sequencing of bacterial 16S rDNA and fungal ITS region

An aliquot of the extracted DNA from each sample was used as a template for amplification. The primers 338F and 806R were used to amplify the V3−V4 hypervariable region of the bacterial 16S rRNA gene (Cui et al., 2019; Huse et al., 2008). The primers ITS1F and ITS2R were used to amplify the ITS1 region of the fungal rDNA (Gardes & Bruns, 1993; White et al., 1990). The PCR products were sequenced on an Illumina MiSeq platform (Illumina, San Diego, CA, USA).

### Bioinformatic analysis

The primer sequences were trimmed after the raw sequences were denoised, sorted and separated using Trimmomatic (Bolger, Lohse & Usadel, 2014). The qualified sequences were clustered into operational taxonomic units (OTUs) at a 97% similarity level using Uparse (Edgar, 2013). The singletons were removed before the downstream analyses. The taxonomic identification of representative sequences for each OTU was determined using the Silva reference database (http://www.arb-silva.de) for the 16S rRNA genes and the Unite reference database (http://unite.ut.ee/index.php) for the ITS using an RDP classifier (Wang et al., 2007). To account for the effects of different sequencing depths, the OTU tables were normalized to identical sequencing depth. The Chao1, Shannon-Wiener, and inverse Simpson indices were calculated to evaluate the richness and diversity of the soil microbial community in mothur (Schloss et al., 2009). All sequences were submitted to the NCBI Sequence Read Archive under SRP216567 and SRP216582.

### Data analyses

Student’s *t*-test was used to examine the differences in soil characteristics, soil molecular biomass, microbial abundance, and alpha diversity between samples collected in urban and suburban park soils. Pearson correlation analysis was used to assess the associations between the abundance and alpha diversity of the microbial communities and environmental factors. These statistical analyses were performed using SPSS 16.0 software (SPSS Inc., Chicago, IL, USA). Differences at *P* < 0.05 were regarded as statistically significant. To meet assumptions of normality, the original data were log transformed prior to analysis when necessary. A similarity analysis (ANOSIM) based on the relative abundance of bacterial or fungal genera was conducted to test the significant difference of the microbial community composition between urban and suburban park soils in the R software package using the vegan library (http://www.r-project.org). To identify the microbial taxa responsible for the community differentiation between urban and suburban park soils, we employed student’s t-test on all of the genera using STAMP software (Parks et al., 2014). The differential genera with false discovery rate-corrected *P* values < 0.05 were identified as indicator genera (Benjamini & Hochberg, 1995). Network analysis was conducted for bacterial and fungal communities based on OTU relative abundances in urban and suburban park soils, yielding a total of 4 networks. Only OTUs detected in 7 out of 10 replicate samples were used for network analysis. Random matrix theory (RMT) was used to identify the appropriate similarity threshold before network construction (Zhou et al., 2010). The cutoff values for the similarity matrix were 0.920 and 0.850 for the bacterial and fungal networks, respectively. Network analysis was performed in Molecular Ecological Network Analyses (MENA) Pipeline (http://ieg2.ou.edu/MENA/) (Deng et al., 2012). Within-module connectivity (*Zi*) and among-module connectivity (*Pi*) were used to sort nodes as peripherals (*Zi* < 2.5 and *Pi* < 0.62), connectors (*Pi* > 0.62), module hubs (*Zi* > 2.5) or network hubs (*Zi* > 2.5 and *Pi* > 0.62) (Deng et al., 2012; Olesen et al., 2007). Principal component analysis (PCA) was conducted to evaluate the differences of soil characteristics between urban and suburban park soils (Canoco 5.0). Redundancy analysis (RDA) was conducted to identify significant factors shaping the structures of the microbial communities at the genus level (Canoco 5.0). Conditional effects of the environmental factors were calculated through the Monte Carlo permutation test (999 permutations) in RDA analysis. Conditional effects represent the amount of additional variation each environmental factor contributes when it is added to the model (Leps & Smilauer, 2003; Ter Braak & Smilauer, 2012). Environmental factors were divided into two sections including soil properties (pH, SOC, total and available N, total and available P, total and available K) and pollutants (available Cu, Pb, Cd, Cr, Ni, Zn, and As). Variation partitioning analysis was used to evaluate the influence of soil properties and pollutants on the soil microbial community structure (Canoco 5.0).

## Results

### Soil characteristics

**Figure 1:**
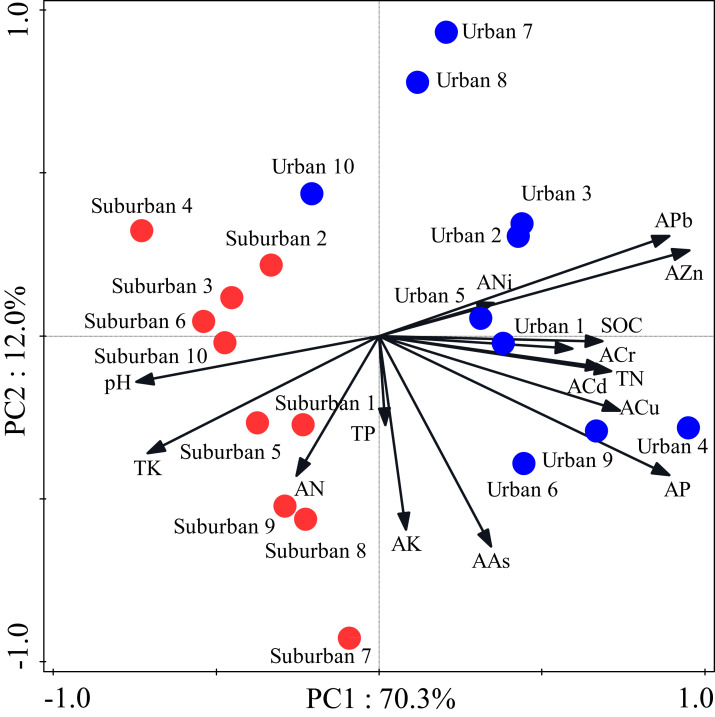
Principal component analysis of the soil characteristics in urban and suburban parks. Blue closed circles: the urban park soil; red closed circles: the suburban park soil.

Most soil characteristics differed significantly between urban and suburban park soils (Table 2). All soil samples were alkaline. The soil pH was significantly higher in suburban park soils than that in urban park soils. The concentrations of SOC, total N, and available P in urban park soils were significantly higher than those in suburban park soils, but total K and available N showed the opposite trend (Table 2). The concentrations of total P and available K in urban and suburban park soils were similar to each other. As expected, the concentrations of soil heavy metals, such as available Pb, Cr, Zn were significantly higher in urban park soils than those in suburban park soils (Table 2). However, the concentrations of available Cu, Cd, Ni, and As in urban and suburban park soils were similar to each other. The PCA results showed a separation between urban and suburban park soils along the first axis (Fig. 1).

**Table 2:** Soil chemical properties of the park soils collected in urban and suburban areas in Shanghai.

Soil properties	Urban park soils	Suburban park soils
pH	7.71 ± 0.07 b	8.25 ± 0.04 a
Soil organic carbon (g kg^−1^)	18.98 ± 1.23 a	14.46 ± 1.23 b
Total nitrogen (g kg^−1^)	1.33 ± 0.09 a	0.93 ± 0.12 b
Total phosphorus (g kg^−1^)	0.70 ± 0.02 a	0.75 ± 0.03 a
Total potassium (g kg^−1^)	18.26 ± 0.38 b	20.89 ± 0.41 a
Available nitrogen (mg kg^−1^)	88.80 ± 5.70 b	114.06 ± 8.44 a
Available phosphorus (mg kg^−1^)	17.33 ± 3.21 a	8.05 ± 1.26 b
Available potassium (mg kg^−1^)	215.80 ± 15.04 a	230.09 ± 18.66 a
Available copper (mg kg^−1^)	10.32 ± 0.77 a	8.66 ± 0.35 a
Available lead (mg kg^−1^)	9.00 ± 0.60 a	4.68 ± 0.29 b
Available cadmium (mg kg^−1^)	0.12 ± 0.02 a	0.07 ± 0.003 a
Available chromium (mg kg^−1^)	0.06 ± 0.04 a	0.008 ± 0.003 b
Available nickel (mg kg^−1^)	0.46 ± 0.03 a	0.39 ± 0.03 a
Available zinc (mg kg^−1^)	11.38 ± 1.33 a	2.99 ± 0.17 b
Available arsenic (mg kg^−1^)	0.18 ± 0.01 a	0.17 ± 0.01 a

**Note:**

a, b means significant differences between soil samples at *P* < 0.05.

### Microbial abundance

**Figure 2:**
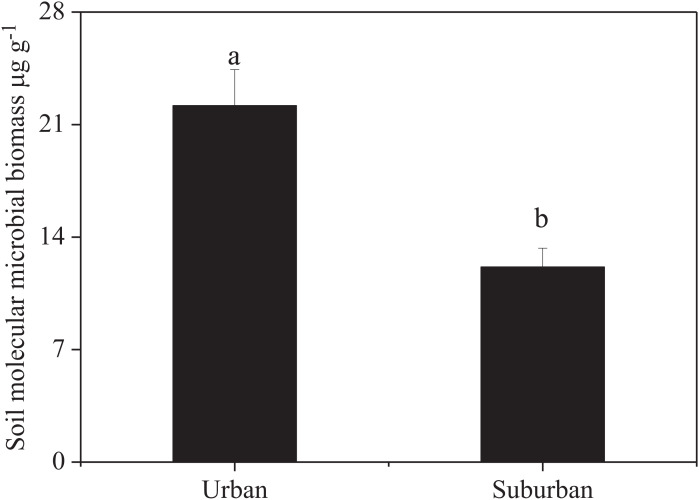
Soil molecular microbial biomass in urban and suburban park soils. Error bars indicate the standard error of the means. a, b means significant differences between soil samples at *P* < 0.05.

**Figure 3:**
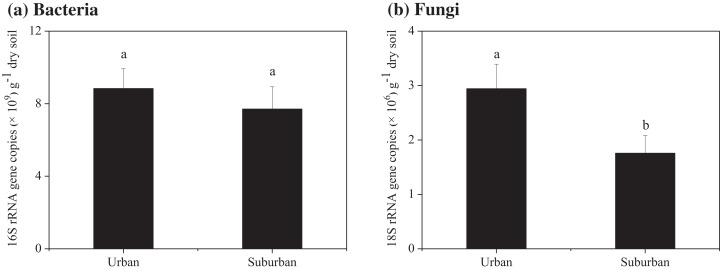
Abundance of the bacterial 16S rRNA (A) and fungal 18S rRNA genes (B) in the park soils. Error bars indicate the standard error of the means. a, b means significant differences between soil samples at *P* < 0.05.

Soil molecular biomass in urban park soils was ~1.8 fold greater than that in suburban park soils (Fig. 2). Compared to suburban park soils, the abundance of fungi in urban park soils significantly increased, while no significant difference was observed in the abundance of bacterial communities in urban and suburban park soils (Fig. 3).

### Microbial alpha diversity, community composition and ecological network

In total, 366,300 bacterial sequences and 1,022,560 fungal sequences were used for analysis. Shannon, Chao1, and inverse Simpson indices were used to compare the levels of microbial alpha diversity. Soils from suburban parks presented a lower fungal Chao1 index and differed significantly from those of urban park soils (Fig. S1). In contrast, the bacterial Chao1 index was similar between the soils collected from urban and suburban parks. No significant difference was observed in the Shannon and inverse Simpson indices of soil bacterial and fungal communities (Fig. S1).

**Figure 4:**
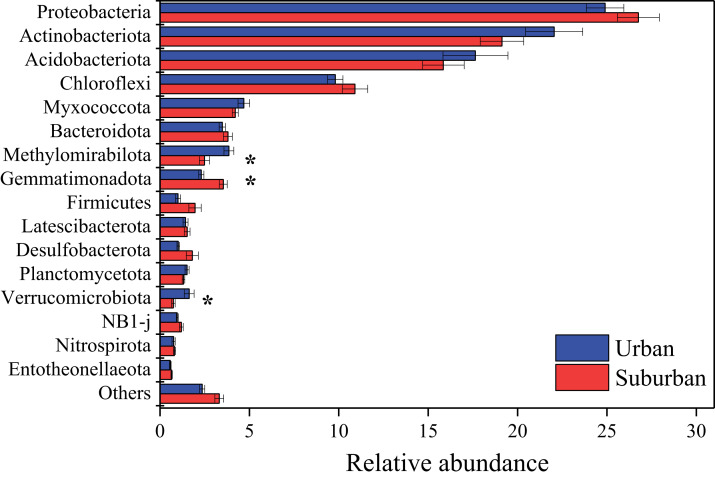
Relative abundances of the bacterial phyla in the park soils. Error bars indicate the standard error of the means. An asterisk (*) means significant differences between soil samples at *P* < 0.05.

Proteobacteria (25.8%), Actinobacteriota (20.6%), Acidobacteriota (16.7%), and Chloroflexi (10.4%) were the predominant bacterial phyla in all soil samples (Fig. 4). The relative abundances of these bacterial phyla were similar between soils collected from urban and suburban parks. In contrast, the relative abundances of several bacterial phyla were significantly different between urban and suburban soils, such as Methylomirabilota, Gemmatimonadota, and Verrucomicrobiota (Fig. 4). Methylomirabilota and Verrucomicrobiota were significantly enriched in urban park soils, while Gemmatimonadota was significantly enriched in suburban park soils. A total of 680 bacterial genera were observed in this study, and 614 bacterial genera shared between urban and suburban park soils (Fig. S2). The most abundant genera (relative abundance >1%) were shown in Fig. S3, including MND1, *Gaiella*, and *Pedomicrobium*. The relative abundances of 53 bacterial genera in urban park soils were significantly different from those in suburban park soils (Table S1). The relative abundance of MND1 in suburban park soils was significantly higher than that in urban park soils. However, the relative abundance of *Gaiella* exhibited the opposite trend. The result of the ANOSIM also revealed significant variations in the bacterial communities between the different sampling areas (R = 0.384, *P* = 0.001). The bacterial sequences were clustered into 3,606 OTUs in this study, and the majority in very low relative abundances (<0.1%) (Table S2).

**Figure 5:**
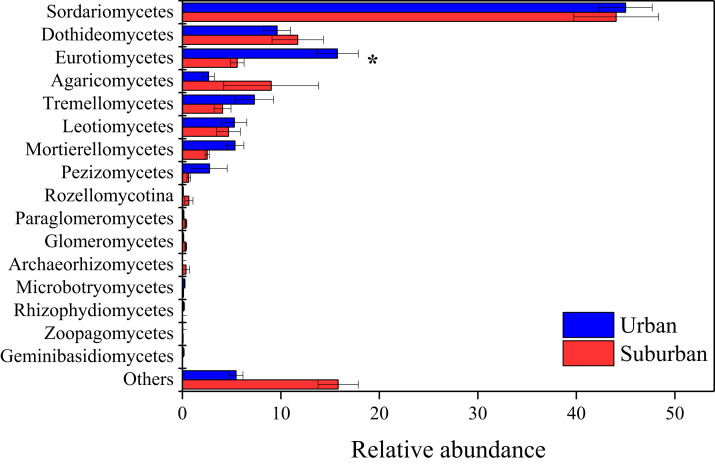
Relative abundances of the fungal classes in the park soils. Error bars indicate the standard error of the means. An asterisk (*) means significant differences between soil samples at *P* < 0.05.

The fungal community in park soils was dominated by sequences assigned to Ascomycota (74.8%), Basidiomycota (15.8%), and Mortierellomycota (4.0%). Sordariomycetes (44.5%), Dothideomycetes (10.7%), Eurotiomycetes (10.7%), Agaricomycetes (5.8%), and Tremellomycetes (5.7%) were the abundant fungal classes in all soil samples (Fig. 5). The relative abundance of Eurotiomycetes was significantly higher in urban park soils compared to that in suburban park soils. A total of 654 fungal genera were observed in this study, and 431 fungal genera shared between urban and suburban park soils (Fig. S2). The most abundant genera (relative abundance > 1%) were shown in Fig. S3, including *Talaromyces*, *Metarhizium*, and *Neocosmospora*. The relative abundances of all the fungal genera in urban and suburban park soils were similar to each other. However, the result of the ANOSIM indicated that fungal community structures differed significantly between the different sampling areas (R = 0.233, *P* = 0.001). The fungal sequences were clustered into 3,234 OTUs in this study. Similar to bacteria, the majority of the fungal OTUs were also in very low relative abundances (<0.1%) (Table S2).

**Figure 6:**
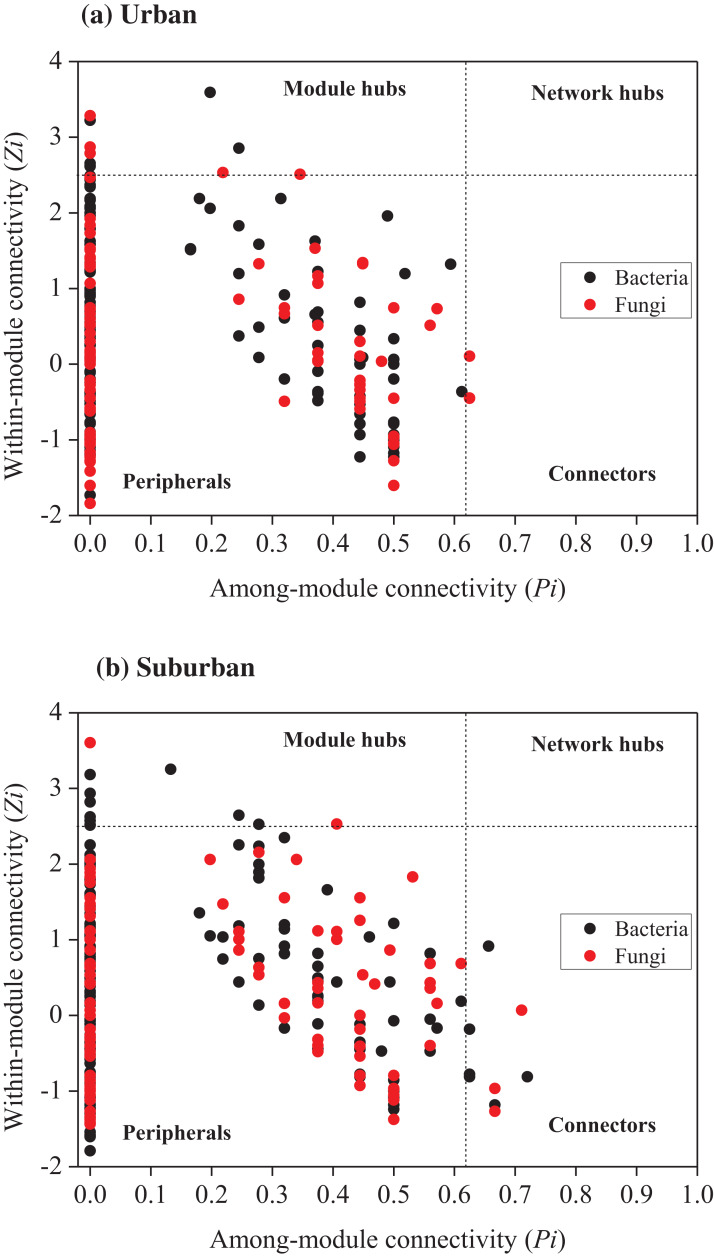
*Zi*-*Pi* plot showing the distribution of OTUs based on their topological roles in networks of bacteria and fungi from urban (A) and suburban park soils (B). Each symbol represented an OTU in the bacterial (black circle) or fungal (red circle) network. The threshold values of *Zi* and *Pi* for categorizing OTUs were 2.5 and 0.62, respectively. Module hubs have *Zi* > 2.5, whereas connectors have *Pi* > 0.62.

Relationships among the bacterial and fungal taxa were estimated by constructing molecular ecological networks for soils collected from urban or suburban parks (Table 3). The bacterial networks with 952 and 945 nodes were constructed from urban and suburban park soils, respectively. The fungal networks with 254 and 222 nodes were constructed from urban and suburban park soils, respectively. R^2^ of power-law for the networks of our study ranged from 0.765 to 0.933, indicating the scale-free property of the networks (Table 3). The modularity values were from 0.736 to 0.877, suggesting that the constructed networks were modular. Furthermore, we determined the topological role of each OTU in microbial networks via RMT-based network analysis (Fig. 6). Regarding the bacterial networks, suburban park soils had more module hubs (13 OTUs) and connectors (7 OTUs) than urban park soils (10 and 0 OTUs, respectively). In urban park soils, the majority of bacterial module hubs were affiliated with Acidobacteriota, Actinobacteriota, and Proteobacteria, while the majority of module hubs and connectors belonged to Chloroflexi, Gemmatimonadota, Myxococcota, Desulfobacterota, and Proteobacteria in the bacterial network of suburban park soils (Table S3). In addition, a decrease in negative interactions in the bacterial network of urban park soils was observed, compared to suburban park soils. Regarding the fungal networks, urban park soils had more module hubs (5 OTUs) than suburban park soils (2 OTUs). Fungal module hubs and connectors in suburban park soils were exclusively affiliated with the specific Ascomycota phylum, while two of the three connectors belonged to Mortierellomycota in the fungal network of urban park soils (Table S3). A moderate decrease in negative interactions in the fungal network of urban park soils was observed, compared to suburban park soils (Table 3).

**Table 3:** Topological properties of the molecular ecological networks of bacterial and fungal communities in the park soils.

Networks	Bacteria		Fungi		
	Urban	Suburban		Urban	Suburban
Total nodes	952	945		254	222
Total links	1,211	1,134		309	352
Negative link percentage (%)	47.5	52.6		46.0	49.7
R^2^ of power-law	0.933	0.907		0.836	0.765
Average degree (avgK)	2.544	2.400		2.433	3.171
Average path distance (GD)	9.427	9.022		7.139	5.940
Average clustering coefficient (avgCC)	0.147	0.155		0.148	0.202
Transitivity	0.346	0.306		0.272	0.287
Modularity	0.865	0.877		0.829	0.736

### Effect of environmental variables on the abundance and composition of microbial communities

**Figure 7:**
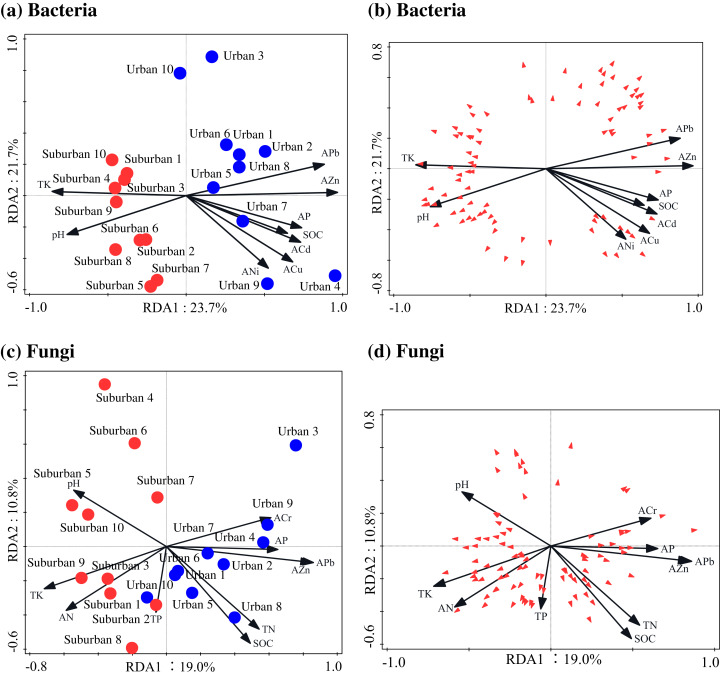
Redundancy analysis used to identify the relationships among the bacterial (A, B) and fungal (C, D) genera (trilateral), soil properties and heavy metals. Blue closed circles: the urban park soil; red closed circles: the suburban park soil.

The Pearson correlation analysis showed that soil molecular microbial biomass was positively correlated with SOC, total N, available Pb, and available Zn, and negatively correlated with soil pH (Table S4). Similar to soil molecular microbial biomass, 18S rRNA gene copies were also positively correlated with SOC and total N (Table S4). The correlation analysis showed that the soil bacterial Chao1 index was negatively correlated with soil available N, total P, and total K, but positively correlated with available Cr and Ni (Table S4). The soil fungal Chao1 index was negatively correlated with soil pH, and positively correlated with SOC, total N, available Pb, and available As (Table S4). The RDA analysis indicated that available Zn was the most important factor shaping the bacterial and fungal communities (Fig. 7; Table 4). In addition to available Zn, available N, pH, and total K were also the primary drivers of fungal community composition.

**Table 4:** Conditional effects of the environmental factors on the community composition of bacteria and fungi determined by the Monte Carlo permutation test in RDA analysis.

Soil chemical properties	Baceria		Soil chemical properties	Fungi	
	Conditional effects (%)	*P*-value		Conditional effects (%)	*P*-value
Avaible Zn	22.4	0.002	Avaible Zn	14.9	0.002
Avaible Cu	9.5	0.076	Avaible N	9.1	0.008
pH	8.7	0.052	pH	8.1	0.010
SOC	6.6	0.132	Total K	7.0	0.040
Total K	6.9	0.084	SOC	6.4	0.066
Avaible Pb	3.7	0.282	Total P	5.5	0.120
Avaible P	3.4	0.366	Avaible P	4.5	0.272
Avaible Ni	2.2	0.604	Avaible Pb	3.8	0.454
Avaible Cd	2.1	0.692	Avaible Cr	3.1	0.604
			Total *N*	2.7	0.730

The relative contributions of soil properties and pollutants to the microbial community variance were assessed by variance partitioning analysis. The results showed that soil properties contributed to 7.9% of the bacterial community variation, pollutants explained 9.3% of the variation, and the interaction between the two explained 17.3% of the variation. In addition, soil properties and pollutants independently explained 18.3% and 0.8% of the total fungal community variance, respectively. The interaction between soil properties and pollutants explained 7.2% of the variance in the fungal community.

## Discussion

Our study used high-resolution molecular techniques to analyze the distribution patterns and drivers of the soil microbial communities in urban parks. In this study, three key results were found. First, compared to suburban park soils, soil molecular biomass and fungal abundance in urban park soils significantly increased. Second, the community composition and networks of both bacteria and fungi were significantly different between urban and suburban park soils. Third, contrary to natural ecosystems, soil available Zn was the most important factor for shaping the structures of the bacterial and fungal communities in park soils.

### Differences in microbial community composition and ecological networks between urban and suburban park soils

Soil molecular biomass in urban park soils was significantly higher than that in suburban park soils, which may be due to the higher fungal abundance in these soils. The alpha diversity of soil microbes in urban and suburban park soils was similar to each other, except for Chao1 index of fungal communities. These results did not support our first hypothesis. Our results indicated that compared to the microbial diversity, soil molecular biomass was more sensitive to environmental changes, and might be used as an indicator of soil quality (Sébastien et al., 2012).

The results of similarity analysis (ANOSIM) revealed that the composition of the microbial communities in urban park soils was significantly different from that in suburban park soils, supporting our second hypothesis. In this study, the main bacterial phyla Proteobacteria, Acidobacteriota, Actinobacteriota, and Chloroflexi were observed in all soil samples. These results were consistent with previous studies conducted in urban ecosystems, indicating that the dominant phyla of bacterial communities in urban soils may be similar (Ramirez et al., 2014; Wang, Wu & Kumari, 2018; Xu et al., 2014; Zhalnina et al., 2015). Moreover, it has been reported that the bacterial phyla Proteobacteria, Acidobacteriota, and Actinobacteriota were also predominant in agricultural soils, forest soils, and even in heavy metal polluted soils (Hemmat-Jou et al., 2018; Sul et al., 2013; Zhang et al., 2017). Thus, the similar relative abundances of these bacterial phyla in urban and suburban park soils may be due to the fact that these phyla can well adapt to various environments. In contrast to the predominant bacterial phyla, the relative abundances of Methylomirabilota, Gemmatimonadota, and Verrucomicrobiota differed significantly among the soils collected in different areas, indicating that these phyla were more sensitive than the predominant bacterial phyla to environmental changes. In this study, the relative abundance of Gemmatimonadota was found to be higher in suburban park soils than those in urban park soils, while the relative abundance of Verrucomicrobiota exhibited the opposite trend. Gemmatimonadota is usually defined as copiotrophic members, and Verrucomicrobiota is generally slow-growing and oligotrophic (Bergmann et al., 2011; Fierer, Bradford & Jackson, 2007; Li et al., 2014). The differences in bacterial communities indicated that compared with soils in suburban parks, soils in urban parks were more conducive to the growth of oligotrophic bacteria, which may be due to their lower concentrations of available N and total K (Li et al., 2014; Wang et al., 2018). Moreover, warming could reduce the abundance of Gemmatimonadota, which may be another explanation for lower relative abundance of Gemmatimonadota in urban park soils (Aislabie & Deslippe, 2013). It has been reported that soil temperature in urban areas was 0.7 °C higher than that in suburban areas because of urban heat island effect (Carreiro et al., 2009). The fungal class Eurotiomycetes was significantly enriched in urban park soils. The most abundant taxa in this class included *Penicillium*, *Aspergillus*, and *Talaromyces*. These genera were also reported as heavy metal tolerant genera in agricultural and forest soils with heavy metal contamination (Abdel-Azeem et al., 2007; Torres-Cruz et al., 2018). Thus, compared to suburban park soils, the higher relative abundances of these genera in urban park soils may be due to their resistance to heavy metals. It should be noted that these genera were also strongly associated with allergic respiratory disease, especially asthma (Abdel-Azeem & Rashad, 2013; Horner et al., 1995). Therefore, from the perspective of human health, more attention should be paid to the increase of Eurotiomycetes in urban park soils in future.

Compared with suburban park soils, nodes and average paths of the bacterial and fungal networks were higher in urban park soils, indicating that the topological roles of the soil microbes were significantly different between urban and suburban park soils. Moreover, the numbers of module hubs and connectors of the bacterial networks were higher in suburban park soils than those in urban park soils. Previous studies have shown that module hubs and connectors of the network may be considered as key species of the communities because they play important roles in maintaining network integrity (Faust & Raes, 2012; Olesen et al., 2007). Losses of these ‘keystone’ nodes may reduce the stability of the microbial community to perturbation (Lu et al., 2013; Olesen et al., 2007). Moreover, four of twenty module hubs and connectors of the bacterial network in suburban park soils belonged to Chloroflexi, the members of which can respire organochlorines (Krzmarzick et al., 2011). Losses of these module hubs and connectors in urban park soils may reduce the ability of soil to purify such organic pollutants. Two OTUs which belonged to the genus *Mortierella* were observed as connectors of the fungal network in urban park soils. It has been reported that the healthy soil microbiome contained more genus *Mortierella* than the microbiomes of *Fusarium* wilt-diseased soil (Yuan et al., 2020). Compared to suburban park soils, the lower relative abundance of the genus *Fusarium* in urban park soils in this study confirmed that the existence of these connectors of the fungal network may improve the ability of urban park soil to resist soil borne pathogens. In addition, compared to the network of suburban park soils, the decrease in negative interactions was observed in the networks of urban park soils, especially the bacterial network, suggesting that antagonistic or competitive interactions decreased in the microbial communities in urban park soils.

### Drivers of soil bacterial and fungal communities in park soils

The soil properties and pollutants in our study had obvious distributions between urban and suburban park soils, which may lead to differences in the distribution of the abundance and composition of the belowground microbial communities. Previous studies showed that heavy metal pollution decreased soil microbial biomass and diversity because of a decrease in the substrate utilization efficiency for microbes subjected to metal stress (Chen et al., 2014; Giller, Witter & Mcgrath, 2009; Xie et al., 2016). In contrast, in this study, compared with suburban park soils, soil molecular biomass and the abundance of fungi were higher in urban park soils, where the contents of heavy metals were higher. A possible reason for our results was that several heavy metals, such as Zn and Cu, were essential for the physiological functioning of soil microbes, and they only became toxic at high concentrations (Kamal, Prasad & Varma, 2010). The results confirmed that increasing metal stress in soils may lead to an increase or decrease in microbial abundance, which depends on the concentrations of heavy metals (Giller, Witter & Mcgrath, 2009).

In our study, the RDA analysis indicated that soil available Zn was the most important factor shaping the bacterial and fungal communities in park soils. A study conducted in Beijing, China, showed that total Zn was closely correlated with bacterial diversity and community composition in urban ecosystems (Hu et al., 2018). Compared to other soil characteristics, Zn played a more substantial role in shaping the soil microbial communities, which may be due to its relatively higher concentration in park soils than natural soils.

In this study, we divided the environmental factors into two groups based on soil properties and pollution factors to determine which are more important in shaping the composition of the microbial community. The results of RDA and variance partitioning analysis showed that the bacterial community in park soils was driven more by shifts in the heavy metals than in the soil pH and nutrients. Moreover, compared to fungi, the bacterial community in park soils was more sensitive to variations in heavy metals.

## Conclusions

This study provides insights into the distributional patterns, co-occurrence relations, and drivers of microbial communities in urban and suburban park soils in Shanghai, and improves our understanding of microbial ecology in urban ecosystems. Soil molecular biomass and fungal abundance in urban park soils were significantly higher than those in suburban park soils. However, the alpha diversity of soil microbes in urban and suburban park soils was similar to each other, except for Chao1 index of fungal communities. These results indicated that compared to the microbial diversity, soil molecular biomass was more sensitive to environmental changes and might be used as an indicator of soil quality in urban ecosystems. The results of similarity analysis (ANOSIM) revealed remarkable differences in the composition of bacterial and fungal communities at the genus level between urban and suburban park soils. The predominant bacterial phyla in park soils were Proteobacteria, Acidobacteriota, Actinobacteriota, and Chloroflexi, and the relative abundances of these phyla in urban and suburban park soils were similar to each other. In contrast, urban park soils were enriched with the phyla Methylomirabilota and Verrucomicrobiota, while the relative abundance of Gemmatimonadota was higher in suburban park soils. Fungal class Eurotiomycetes was significantly enriched in urban park soils, possibly due to their resistance to heavy metals. In addition, the topological roles of the soil microbes were significantly different between urban and suburban park soils. Compared with suburban park soils, Acidobacteriota bacterium and Mortierellomycota fungus played more important roles in the ecological networks of urban park soils. Both soil properties and pollutants had significant impacts on the abundance and composition of microbial communities. Compared with the fungal community, bacteria were more sensitive to heavy metal pollution stress in park soils. Soil available Zn was the most important factor shaping the structures of both bacterial and fungal communities in this study. In conclusion, there were significant differences in the microbial communities between urban and suburban park soils, and soil available Zn played an important part in shaping the structures of both the bacterial and fungal communities in park soils in Shanghai.

## Supplemental Information

10.7717/peerj.11231/supp-1 Supplemental Information 1 Raw data.

10.7717/peerj.11231/supp-2 Supplemental Information 2 Diversity index of the bacterial and fungal communities in the park soils. Error bars indicate the standard error of the means. a, b means significant differences between soil samples at *P* < 0.05.

10.7717/peerj.11231/supp-3 Supplemental Information 3 Venn diagram illustrating the numbers of unique and shared bacterial (a) and fungal (b) genera in urban and suburban park soils.

10.7717/peerj.11231/supp-4 Supplemental Information 4 Relative abundances of the dominant bacterial (a) and fungal (b) genera (relative abundances > 1%) in urban and suburban park soils. Error bars indicate the standard error of the means. * means significant differences between soil samples at P < 0.05.

10.7717/peerj.11231/supp-5 Supplemental Information 5 Bacterial genera with different relative abundance in urban and suburban park soils.

10.7717/peerj.11231/supp-6 Supplemental Information 6 Number of OTUs with different relative abundance in urban and suburban park soils.

10.7717/peerj.11231/supp-7 Supplemental Information 7 Module hubs and connectors in the molecular ecological networks of bacterial and fungal communities in urban and suburban park soils.

10.7717/peerj.11231/supp-8 Supplemental Information 8 Correlation coefficients between the abundance and alpha diversity of microbial community and environmental factors.

## Additional Information and Declarations

### DNA Deposition

The following information was supplied regarding the deposition of DNA sequences: All sequences are available in the NCBI Sequence Read Archive: SRP216567 and SRP216582.

### Data Availability

The following information was supplied regarding data availability: The raw measurements are available in the Supplemental File.
