# Supplementary figures and images for: Diversity patterns and drivers of soil microbial communities in urban and suburban park soils of Shanghai, China
Source: PeerJ. 2021 Apr 15;9:e11231. doi: 10.7717/peerj.11231 (PMC8053383; doi:10.7717/peerj.11231)

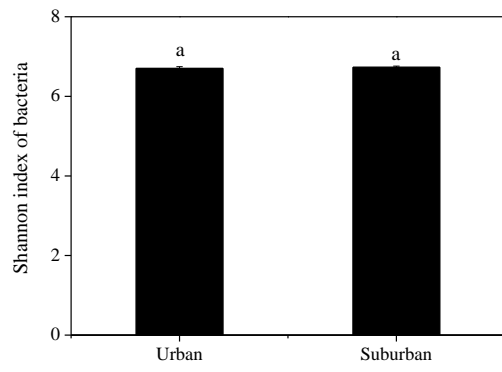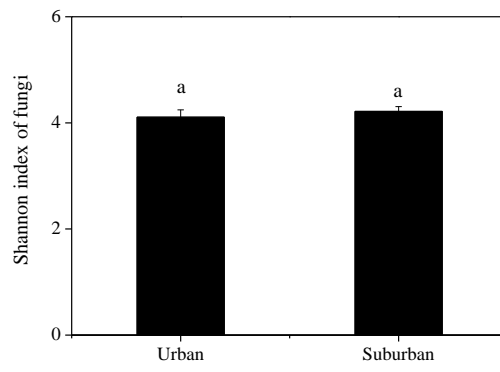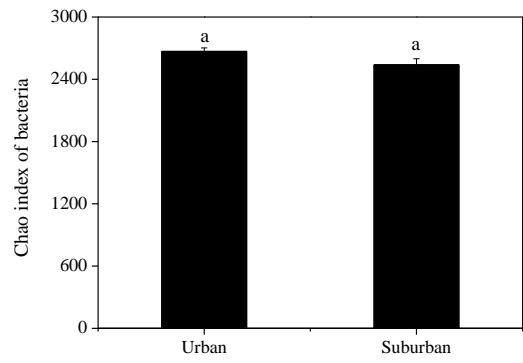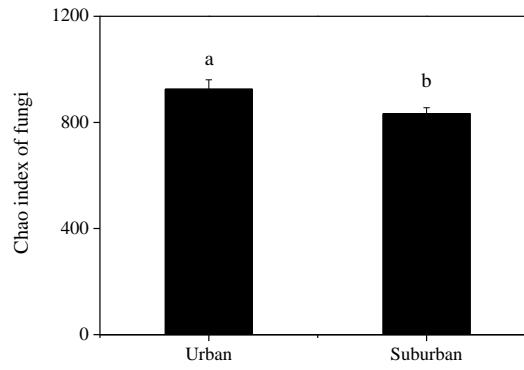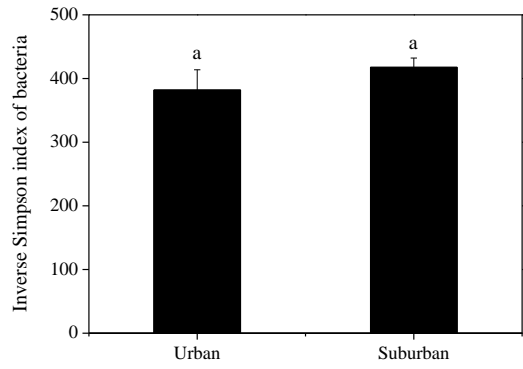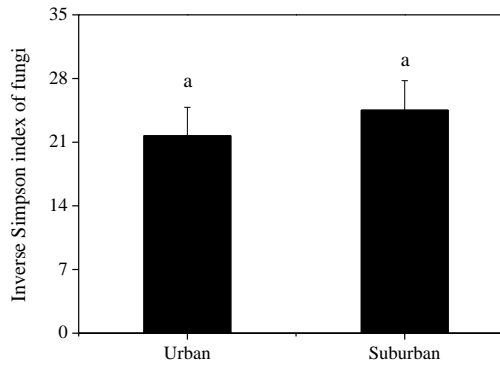

Supplement: Supplemental Information 2 — Error bars indicate the standard error of the means. a, b means significant differences between soil samples at P < 0.05. [file peerj-09-11231-s002.pdf]

**(a) Bacteria**

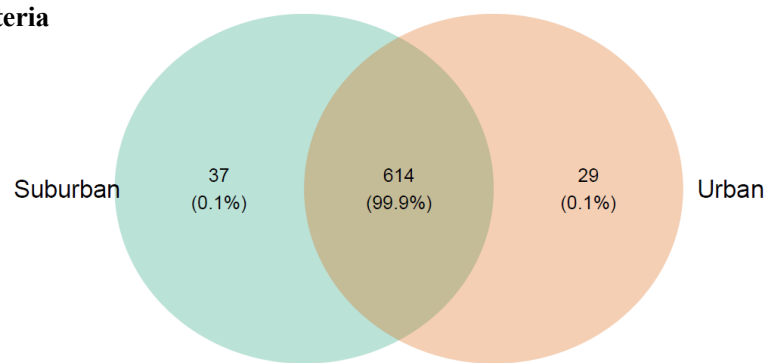

**(b) Fungi**

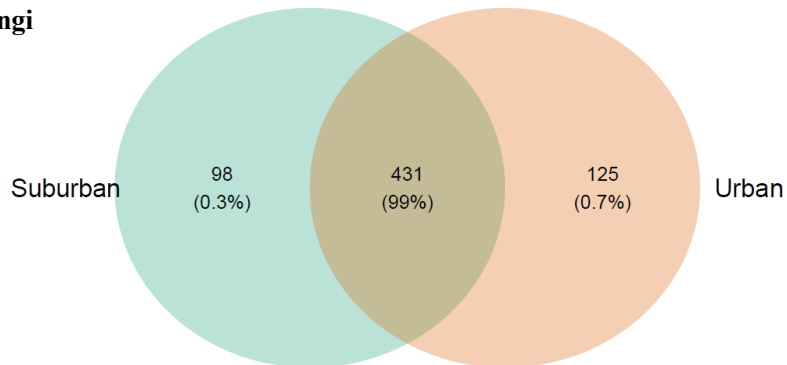

Supplement: Supplemental Information 3 [file peerj-09-11231-s003.pdf]

### (a) Bacteria

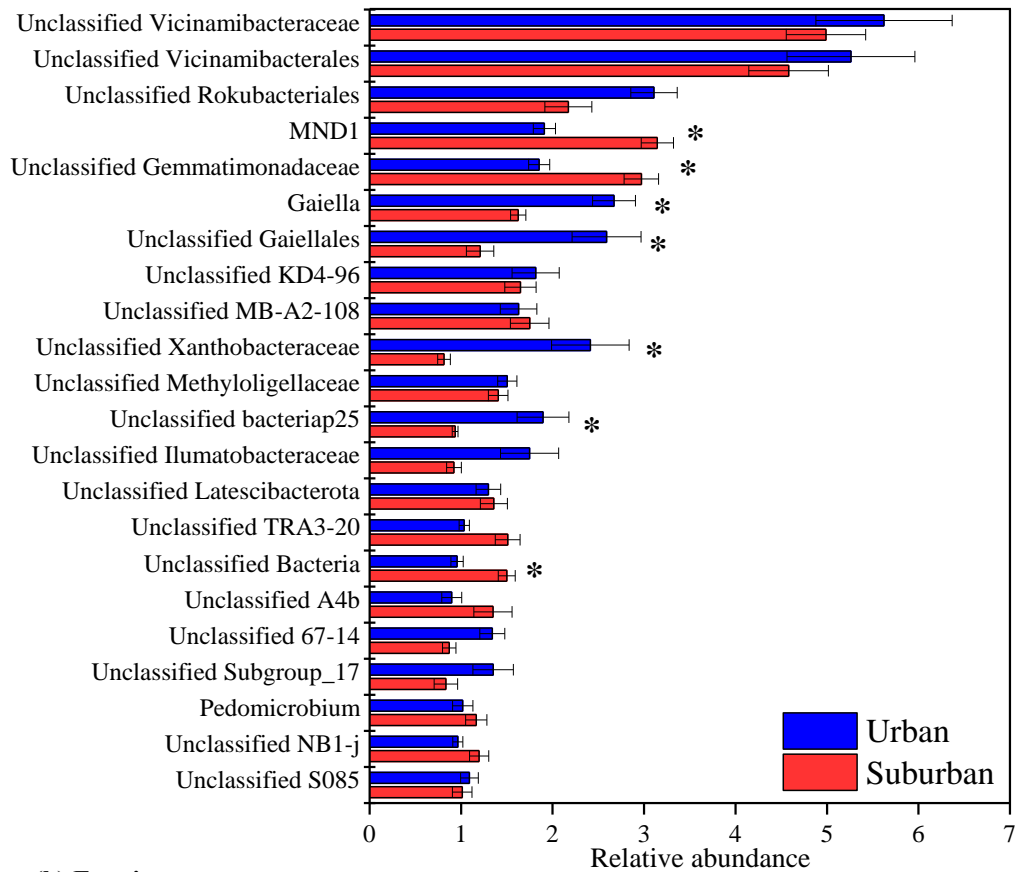

### (b) Fungi

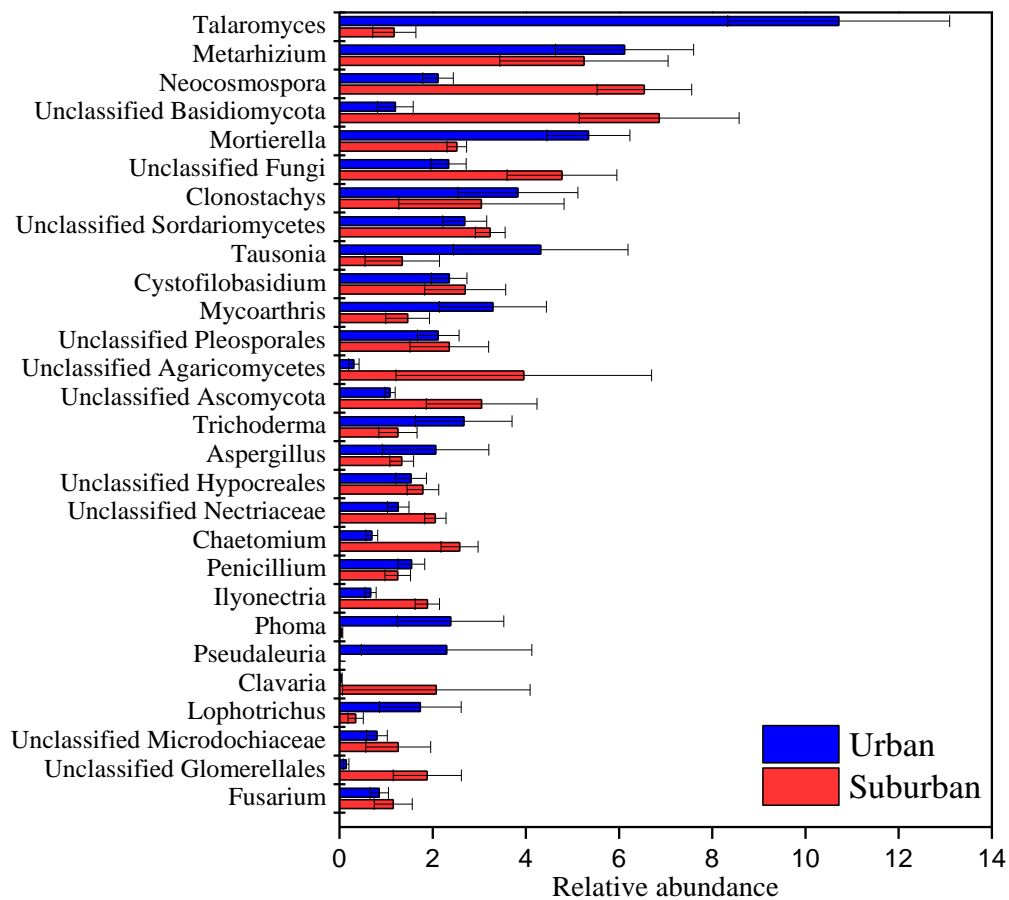

Supplement: Supplemental Information 4 — Error bars indicate the standard error of the means. * means significant differences between soil samples at P < 0.05. [file peerj-09-11231-s004.pdf]
