# Supplementary material for: Diversity patterns and drivers of soil microbial communities in urban and suburban park soils of Shanghai, China
Source: PeerJ. 2021 Apr 15;9:e11231. doi: 10.7717/peerj.11231 (PMC8053383; doi:10.7717/peerj.11231)
Supplement: Supplemental Information 5 [file peerj-09-11231-s005.docx]

**Table S1** Bacterial genera with different relative abundance in urban and suburban park soils.

| Bacterial genera | Urban park soils | Suburban park soils |
| --- | --- | --- |
| MND1 | 1.910±0.121 b | 3.145±0.176 a |
| Unclassified Gemmatimonadaceae | 1.853±0.115 b | 2.970±0.188 a |
| Gaiella | 2.672±0.235 a | 1.624±0.084 b |
| Unclassified Gaiellales | 2.590±0.376 a | 1.208±0.150 b |
| Unclassified Xanthobacteraceae | 2.413±0.425 a | 0.814±0.069 b |
| Unclassified bacteriap25 | 1.895±0.283 a | 0.935±0.032 b |
| Unclassified Bacteria | 0.957±0.067 b | 1.499±0.093 a |
| Unclassified Actinomarinales | 0.509±0.048 b | 1.058±0.100 a |
| Unclassified IMCC26256 | 0.939±0.058 a | 0.592±0.038 b |
| Unclassified Steroidobacteraceae | 0.553±0.035 b | 0.899±0.062 a |
| Unclassified Alphaproteobacteria | 0.746±0.042 a | 0.476±0.039 b |
| Unclassified WX65 | 0.713±0.054 a | 0.311±0.029 b |
| Candidatus_Xiphinematobacter | 0.960±0.235 a | 0.041±0.018 b |
| Unclassified Azospirillales | 0.266±0.022 b | 0.623±0.041 a |
| Ramlibacter | 0.295±0.022 b | 0.518±0.055 a |
| Unclassified Entotheonellaceae | 0.501±0.039 a | 0.265±0.024 b |
| Unclassified Nocardioidaceae | 0.107±0.029 b | 0.643±0.158 a |
| Ellin6067 | 0.268±0.019 b | 0.429±0.021 a |
| Mycobacterium | 0.402±0.041 a | 0.148±0.018 b |
| Candidatus_Entotheonella | 0.076±0.009 b | 0.385±0.040 a |
| Unclassified B1-7BS | 0.384±0.043 a | 0.050±0.012 b |
| Candidatus_Alysiosphaera | 0.252±0.020 a | 0.157±0.014 b |
| Micromonospora | 0.122±0.016 b | 0.222±0.025 a |
| Ruminococcus | 0.072±0.017 b | 0.260±0.046 a |
| Unclassified RBG-13-54-9 | 0.222±0.025 a | 0.109±0.016 b |
| Amaricoccus | 0.222±0.025 a | 0.095±0.010 b |
| Skermanella | 0.073±0.009 b | 0.217±0.024 a |
| Unclassified Hyphomicrobiaceae | 0.163±0.014 a | 0.110±0.008 b |
| AKYG587 | 0.173±0.017 a | 0.085±0.006 b |
| Desulfuromonas | 0.059±0.014 b | 0.189±0.031 a |
| 966-1 | 0.210±0.043 a | 0.002±0.001 b |
| Unclassified Zixibacteria | 0.047±0.011 b | 0.138±0.023 a |
| Citrifermentans | 0.032±0.006 b | 0.133±0.022 a |
| Candidatus_Udaeobacter | 0.144±0.028 a | 0.020±0.005 b |
| Unclassified Acidobacteriae | 0.049±0.007 b | 0.100±0.014 a |
| Nakamurella | 0.130±0.030 a | 0.019±0.004 b |
| Fictibacillus | 0.029±0.006 b | 0.119±0.014 a |
| Unclassified Desulfuromonadia | 0.030±0.009 b | 0.105±0.019 a |
| Unclassified Roseiflexaceae | 0.023±0.007 b | 0.101±0.018 a |
| Rubrobacter | 0.110±0.030 a | 0.009±0.004 b |
| Flavisolibacter | 0.035±0.004 b | 0.081±0.011 a |
| Edaphobaculum | 0.075±0.008 a | 0.040±0.005 b |
| Vicinamibacter | 0.037±0.005 b | 0.072±0.007 a |
| Unclassified Eel-36e1D6 | 0.029±0.004 b | 0.074±0.008 a |
| JTB255_marine_benthic_group | 0.068±0.009 a | 0.007±0.003 b |
| Unclassified KD3-93 | 0.038±0.007 a | 0.013±0.003 b |
| Urania-1B-19_marine_sediment_group | 0.040±0.007 a | 0.003±0.002 b |
| Tumebacillus | 0.004±0.001 b | 0.027±0.006 a |
| Thioalkalispira-Sulfurivermis | 0.001±0.001 b | 0.018±0.005 a |
| Unclassified Thermomicrobiaceae | 0.002±0.001 b | 0.014±0.003 a |
| wb1-P19 | 0.013±0.004 a | 0.000±0.000 b |
| Unclassified Lineage_IV | 0.010±0.002 a | 0.002±0.001 b |
| Anaerosolibacter | 0.001±0.001 b | 0.010±0.002 a |

a, b means significant differences between soil samples at P < 0.05.
