# Supplementary material for: Diversity patterns and drivers of soil microbial communities in urban and suburban park soils of Shanghai, China
Source: PeerJ. 2021 Apr 15;9:e11231. doi: 10.7717/peerj.11231 (PMC8053383; doi:10.7717/peerj.11231)
Supplement: Supplemental Information 6 [file peerj-09-11231-s006.docx]

**Table S2** Number of OTUs with different relative abundance in urban and suburban park soils.

| Relative abundance of OTU (%) | Number of bacterial OTUs | | | Number of fungal OTUs | |
| --- | --- | --- | --- | --- | --- |
|  | urban park soils | suburban park soils | urban park soils | | suburban park soils |
| >10 | 0 | 0 | 1.5±0.3 a | | 1.0±0.3 a |
| 1~10 | 3.1±0.7 a | 2.0±0.3 a | 16.4±0.8 a | | 18.3±1.5 a |
| 0.1~1 | 222.4±4.8 a | 228.7±3.4 a | 83.0±5.9 a | | 90.4±3.5 a |
| <0.1 | 1897.1±22.4 a | 1833.8±41.7 a | 681.4±28.1 a | | 617.6±16.6 a |

a, b means significant differences between soil samples at P < 0.05.
