# Supplementary material for: Diversity patterns and drivers of soil microbial communities in urban and suburban park soils of Shanghai, China
Source: PeerJ. 2021 Apr 15;9:e11231. doi: 10.7717/peerj.11231 (PMC8053383; doi:10.7717/peerj.11231)
Supplement: Supplemental Information 7 [file peerj-09-11231-s007.docx]

**Table S3** Module hubs and connectors in the molecular ecological networks of bacterial and fungal communities in urban and suburban park soils.

| Ecological networks | Type of points | OTU ID | Phylum/classes | Lowest taxonomic rank |
| --- | --- | --- | --- | --- |
| Networks of bacterial communities in urban park soils | Module Hubs | OTU1526 | Acidobacteriota/ Vicinamibacteria | c__Vicinamibacteria |
|  |  | OTU3516 | Acidobacteriota/ Vicinamibacteria | o__Vicinamibacterales; |
|  |  | OTU1019 | Acidobacteriota/ Vicinamibacteria | o__Vicinamibacterales; |
|  |  | OTU2368 | Actinobacteriota/ Actinobacteria | f__Intrasporangiaceae |
|  |  | OTU717 | Actinobacteriota/ Thermoleophilia | o__Solirubrobacterales |
|  |  | OTU2939 | Actinobacteriota/ Acidimicrobiia | f__Ilumatobacteraceae |
|  |  | OTU3091 | Proteobacteria/ Alphaproteobacteria | f__Hyphomonadaceae |
|  |  | OTU3220 | Proteobacteria/ Gammaproteobacteria | f__Sutterellaceae |
|  |  | OTU186 | Myxococcota/ Polyangia | o__Polyangiales |
|  |  | OTU123 | Chloroflexi/ Chloroflexia | f__Roseiflexaceae |
| Networks of bacterial communities in suburban park soils | Module Hubs | OTU2111 | Chloroflexi/ Dehalococcoidia | c__Dehalococcoidia |
|  |  | OTU1582 | Chloroflexi/ Dehalococcoidia | c__Dehalococcoidia |
|  |  | OTU3043 | Chloroflexi/ JG30-KF-CM66 | c__JG30-KF-CM66 |
|  |  | OTU635 | Gemmatimonadota/ Gemmatimonadetes | f__Gemmatimonadaceae |
|  |  | OTU268 | Gemmatimonadota/ Gemmatimonadetes | g__Gemmatimonas |
|  |  | OTU2375 | Myxococcota/ bacteriap25 | c__bacteriap25 |
|  |  | OTU1525 | Myxococcota/ Polyangia | f__Sandaracinaceae |
|  |  | OTU2365 | Desulfobacterota/ Desulfuromonadia | c__Desulfuromonadia |
|  |  | OTU2306 | Desulfobacterota/ Desulfobulbia | f__Desulfocapsaceae |
|  |  | OTU2929 | Proteobacteria/ Gammaproteobacteria | f__Nitrosomonadaceae |
|  |  | OTU3547 | Proteobacteria/ Gammaproteobacteria | f__Nitrosomonadaceae |
|  |  | OTU3516 | Acidobacteriota/ Vicinamibacteria | o__Vicinamibacterales |
|  |  | OTU238 | Methylomirabilota/ Methylomirabilia | o__Rokubacteriales |
|  | Connectors | OTU2013 | Chloroflexi/ Dehalococcoidia | c__Dehalococcoidia |
|  |  | OTU2638 | Myxococcota/ Myxococcia | g__Anaeromyxobacter |
|  |  | OTU70 | Proteobacteria/ Alphaproteobacteria | g__Bosea |
|  |  | OTU3019 | Acidobacteriota/ Acidobacteriae | g__Bryobacter |
|  |  | OTU888 | Actinobacteriota/ Actinobacteria | g__Cellulomonas |
|  |  | OTU1176 | Actinobacteriota/ Acidimicrobiia | f__Ilumatobacteraceae |
|  |  | OTU757 | Patescibacteria/ Saccharimonadia | o__Saccharimonadales |
| Networks of fungal communities in urban park soils | Module Hubs | OTU1658 | Ascomycota/ Dothideomycetes | g__Massarina |
|  |  | OTU1699 | Ascomycota/ Dothideomycetes | g__Paraconiothyrium |
|  |  | OTU2870 | Ascomycota/ Sordariomycetes | g__Ramophialophora |
|  |  | OTU881 | Ascomycota/ Sordariomycetes | g__Acremonium |
|  |  | OTU3241 | Ascomycota/ Leotiomycetes | g__Bisporella |
|  | Connectors | OTU499 | Ascomycota/ Dothideomycetes | g__Wiesneriomyces |
|  |  | OTU2528 | Mortierellomycota/ Mortierellomycetes | g__Mortierella |
|  |  | OTU1410 | Mortierellomycota/ Mortierellomycetes | g__Mortierella |
| Networks of fungal communities in suburban park soils | Module Hubs | OTU2324 | Ascomycota/ Sordariomycetes | g__Emericellopsis |
|  |  | OTU1124 | Ascomycota/ Sordariomycetes | g__Trichoderma |
|  | Connectors | OTU442 | Ascomycota/ Sordariomycetes | g__Gibellulopsis |
|  |  | OTU2179 | Ascomycota/ Sordariomycetes | g__Chaetomium |
|  |  | OTU464 | Ascomycota/ Dothideomycetes | f__Didymellaceae |
