# Supplementary material for: Diversity patterns and drivers of soil microbial communities in urban and suburban park soils of Shanghai, China
Source: PeerJ. 2021 Apr 15;9:e11231. doi: 10.7717/peerj.11231 (PMC8053383; doi:10.7717/peerj.11231)
Supplement: Supplemental Information 8 [file peerj-09-11231-s008.docx]

**Table S4**. Correlation coefficients between the abundance and alpha diversity of microbial community and environmental factors.

| Soil characteristics | Soil molecular microbial biomass | Bacterial 16S rRNA gene copies | Fungal 18S rRNA gene copies | Shannon index of bacterial community | Chao1 index of bacterial community | Inverse Simpson index of bacterial community | Shannon index of fungal community | Chao1 index of fungal community | Inverse Simpson index of fungal community |
| --- | --- | --- | --- | --- | --- | --- | --- | --- | --- |
| pH | **-0.600**** | -0.108 | -0.320 | 0.099 | -0.378 | 0.153 | -0.029 | **-0.539*** | 0.065 |
| SOC | **0.649**** | 0.243 | **0.617**** | 0.003 | -0.032 | 0.027 | 0.424 | **0.775**** | 0.314 |
| Total N | **0.512*** | 0.024 | **0.497*** | -0.263 | -0.153 | -0.213 | 0.048 | **0.509*** | 0.138 |
| Available N | -0.225 | -0.349 | -0.053 | -0.201 | **-0.594**** | -0.024 | **0.583**** | 0.109 | 0.377 |
| Total P | -0.252 | 0.147 | -0.201 | -0.400 | **-0.515*** | -0.303 | 0.063 | -0.111 | -0.098 |
| Available P | 0.126 | 0.092 | 0.136 | -0.192 | 0.148 | -0.261 | 0.038 | 0.305 | 0.008 |
| Total K | -0.401 | -0.404 | -0.287 | -0.078 | **-0.644**** | 0.083 | 0.246 | -0.148 | 0.351 |
| Available K | -0.252 | -0.198 | -0.066 | 0.091 | 0.141 | -0.013 | 0.222 | -0.024 | 0.315 |
| Available Cu | 0.148 | 0.139 | 0.220 | 0.015 | 0.245 | -0.053 | 0.119 | 0.345 | -0.047 |
| Available Pb | **0.459*** | 0.203 | 0.246 | -0.246 | 0.259 | -0.333 | -0.059 | **0.462*** | -0.105 |
| Available Cd | 0.241 | 0.325 | 0.199 | -0.270 | 0.015 | -0.287 | 0.067 | 0.305 | -0.067 |
| Available Cr | 0.145 | 0.078 | 0.236 | 0.361 | **0.462*** | 0.313 | -0.010 | 0.166 | -0.169 |
| Available Ni | 0.248 | 0.360 | 0.146 | **0.465*** | **0.543*** | 0.341 | 0.247 | 0.400 | 0.072 |
| Available Zn | **0.514*** | 0.323 | 0.414 | -0.228 | 0.295 | -0.324 | -0.084 | 0.384 | -0.148 |
| Available As | 0.118 | -0.293 | 0.200 | 0.075 | -0.184 | 0.171 | **0.542*** | **0.584**** | **0.566**** |

**^**^** *P* < 0.01; **^*^** *P* < 0.05; n=20 (number of samples used to calculate correlation coefficients).
